# Centenary Dinner for the Bristol Medico-Chirurgical Journal

**Published:** 1983-07

**Authors:** H. M. Norman


### Centenary Dinner for the Bristol Medico-Chirurgical Journal

As a finale to the 1982–83 session of the Society and to celebrate the centenary of the *Journal*, a dinner was held. The President, Mr. H. K. Bourns and his wife received the 78 members and guests in the Red Lodge, Park Row, Bristol accompanied by the Guests of Honour, Professor Sir James Fraser Bt., President of the Royal College of Surgeons, Edinburgh, and Lady Maureen Fraser. After a short reception the assembled company repaired to that distinctive and noble hall, the home of the Bristol Savages, the Wigwam. Here they enjoyed a very good meal, including salmon mayonnaise and strawberries and cream with liberal Liebfraumilch.

After toasting the Queen the President introduced Sir James Fraser, Currently Postgraduate Dean at Edinburgh University and formerly Professor of Surgery at Southampton and Editor of the *Annals of the Royal College of Surgeons of Edinburgh.* Sir James entertained the diners to an amusing account of the editor's job pointing out that his journal was a mere youngster, having been founded in 1955 and he paid tribute to the Society's *Journal* which had now survived 100 years.

In thanking Sir James Mr. Michael Wilson, the Editor and a Green Feather member of the Savages, gave a brief history of the Savages and the Wigwam. He went on to discuss the original number in which the first two papers argued for and against Phthisis being a contagious disease. He said that the *Journal* was 'The world's window on Bristol' and that our articles are indexed in the Index Medicus. He likened his new hobby as Editor to his old hobby of gardening. It was necessary in the garden of the journal to grow mainly vegetables, articles that were nutritious or had high roughage content but one needed also flowers – a pleasure to read but low clinical content., He awarded the title of Prize Orchid to Dr. John Burton's article 'Dermatological DIY'

As epilogue to a delightful evening Mr. Nigel Dodd and Mr. Christopher Northam, two Blue Feather members of the Savages, gave a short recital on two pianos awakening the somnolent with Falla's *Ritual Fire Dance*, followed by the *Blue Danube* with tapping of feet and a number of other favourites old and new.

It was well after 11 o'clock when the President thanked speakers, pianists and diners for a memorable evening and all then departed for bed.

H. M. NORMAN

